# Early Intervention, Parent Talk, and Pragmatic Language in Children With Hearing Loss

**DOI:** 10.1542/peds.2020-0242F

**Published:** 2020-11

**Authors:** Christine Yoshinaga-Itano, Allison L. Sedey, Craig A. Mason, Mallene Wiggin, Winnie Chung

**Affiliations:** a Institute of Cognitive Science, University of Colorado-Boulder, Boulder, Colorado;; b Colorado School for the Deaf and the Blind, Colorado Springs, Colorado;; c School of Learning and Teaching, University of Maine, Orono, Maine;; d Centers for Disease Control and Prevention, Atlanta, Georgia

## Abstract

**BACKGROUND AND OBJECTIVES:**

Pragmatic language skills form the foundation for conversational competence, whereas deficits in this area are associated with behavioral problems and low literacy skills. Children who are deaf or hard of hearing demonstrate significant delays in this critical area of language. Our purpose with this research was to identify variables associated with pragmatic language ability in children who are deaf or hard of hearing.

**METHODS:**

This was a longitudinal study of 124 children with bilateral hearing loss between 4 and 7 years of age living in Colorado. As part of a comprehensive speech and language assessment, pragmatic language skills were evaluated annually by using the Pragmatics Checklist.

**RESULTS:**

The children’s pragmatic skills increased significantly with age. Higher levels of pragmatic language ability at 7 years of age were predicted by (1) meeting Early Hearing Detection and Intervention 1–3–6 guidelines (hearing screening by 1 month, identification of hearing loss by 3 months, and receiving intervention by 6 months of age), (2) greater quantity of parent talk, (3) higher nonverbal intelligence, (4) lesser degrees of hearing loss, and (5) higher maternal education.

**CONCLUSIONS:**

With the findings of this study, we underscore the importance of pediatricians and other health care professionals counseling parents about the value of adherence to the Early Hearing Detection and Intervention 1–3–6 guidelines with regard to intervention outcomes. The strong association between amount of child-directed parent talk in the first 4 years of life and pragmatic language outcomes at 7 years of age emphasizes the need for professionals to encourage parents to talk to their children as much as possible.

The pragmatic aspects of language can be defined as the rules governing the use of language in context.^1^ This social use of language includes 3 major skills: (1) using language for different reasons (eg, greeting, informing, requesting), (2) adapting language in response to the listener or situation, and (3) following conversational rules (eg, turn taking, staying on topic, clarifying).^2^ Typical development of these skills starts in infancy, and foundational competence is demonstrated by 8 years of age.^3^ In contrast, children who are deaf or hard of hearing have been found to have delayed or deviant pragmatic skill development.^4–9^ Researchers have hypothesized that these delays are due to insufficient access to daily discourse and the underlying linguistic structures that support it. Pragmatic language competence is critical in face-to-face communication, and better pragmatic language skills also have been associated with success in educational settings.^10–12^ In contrast, deficits in pragmatic language ability have been associated with lower levels of literacy^13–15^ and an increased incidence of behavioral problems.^16,17^

Although there has been an increase in research on pragmatic language development in school-aged children (with and without hearing loss) over the past decade,^6,18–23^ little is known about what child and family characteristics are associated with better pragmatic language outcomes, particularly in children who are deaf or hard of hearing. Researchers in previous studies have examined predictors of other aspects of language, such as vocabulary development, and have reported a variety of child factors to be related to better outcomes. These include lesser degrees of hearing loss^24–28^ and early enrollment in intervention.^27,29^ Parent variables, such as higher levels of maternal education, also have been associated with improved language outcomes for children who are deaf or hard of hearing.^24,30,31^ In studies involving children who are hearing, greater quantity of parent talk has been associated with better vocabulary outcomes.^32–34^ However, little is known regarding the impact of parent talk on pragmatic language development specifically, particularly in children who are deaf or hard of hearing.

Considering the importance of pragmatic language ability, and the delays that persist for children who are deaf or hard of hearing, analyzing predictors and longitudinal development of this aspect of language may be used to identify factors that can be modified early in life to reduce or prevent delays in later childhood. Our purpose with this study was to identify factors associated with pragmatic language performance and growth in pragmatics skills over time. Specifically, the impact of the following child and parent characteristics was examined: (1) child’s chronological age; (2) child’s nonverbal intelligence quotient (IQ); (3) degree of hearing loss; (4) meeting the Early Hearing Detection and Intervention (EHDI) guidelines of screening, diagnosis, and early intervention by 1, 3, and 6 months of age, respectively; (5) mother’s level of education; and (6) quantity of child-directed parent talk.

## METHODS

### Participants

This was a longitudinal study of 124 children with bilateral, prelingual hearing loss from 3 years, 9 months to 7 years, 3 months of age (mean = 5 years, 6 months). All participants lived in Colorado, had hearing parents, and were from homes in which the primary language was spoken English. Sign language was used consistently in conjunction with spoken language by 16% of the families. During the birth-to-3-year period, the families received early intervention services (1 hour per week) from the Colorado Home Intervention Program. One of the primary components of this program for all families involved teaching parents strategies to maximize their child’s communication skills. See Table 1 for a summary of the participants’ demographic characteristics.

The majority (90%) of the children had no additional disabilities thought to interfere with speech or language development. The remaining 10% reported additional disabilities ranging from sensorimotor integration issues to cognitive delay. The children’s nonverbal IQs ranged from 58 to 140 (mean = 103.1; SD = 16.8) on the basis of the Leiter International Performance Scale-Revised.^35^ Only 4% of the children scored >2 SD below the test mean of 100.

The children’s degree of hearing loss ranged from mild to profound, and all used either hearing aids and/or cochlear implants (see Table 2 for details). Slightly less than half (46%) of the children met the EHDI hearing screening by 1 month, confirmation of hearing loss by 3 months, and intervention by 6 months of age (1–3–6) guidelines.

This project was approved by the Institutional Review Board at the University of Colorado Boulder. All families provided written informed consent.

### Procedures

The children participated in a comprehensive speech and language assessment. They were assessed annually from the ages of 4 to 7 years within 3 months of their birthday. Four annual assessments were conducted for children who started the study at 4 years of age and who turned 7 within the time period of the study.

Because some children entered the study at 5, 6, or 7 years of age, and some were not yet 7 when the study ended, the number of assessments contributed per child ranged from 1 to 4 (mean = 2.4; total number of assessments collected = 302). See further details in Table 3. All testing was conducted by a speech pathologist, psychologist, or teacher of the deaf in a quiet room in the child’s school or a library meeting room.

#### Outcome Variable

The children’s pragmatic language skills were measured by using the Pragmatics Checklist,^5^ a parent-report instrument designed to examine children’s use of 45 different pragmatic language skills (eg, giving directions, offering opinions, requesting clarification, etc). For each item, parents indicated if their child demonstrated the skill and, if so, whether they did so nonverbally, using 1- to 3-word phrases, or using sentences of ≥4 words. Children’s scores were calculated on the basis of the proportion of items they demonstrated when using sentences of 4 words or more. To ease interpretation, this proportion was rescaled to a range of 0 to 10, indicating the corresponding decile (with fractional values) of items the child demonstrated using sentences containing ≥4 words. For example, a score of 6.8 indicates that a child demonstrated 68% of the items when using sentences of ≥4 words.

#### Predictor Variables

The chronological age of the child in months at the time of each assessment was used to assess growth in pragmatic language skills over time. Nonverbal cognitive ability was assessed by using the Leiter International Performance Scale-Revised,^35^ and the full-scale IQ score was entered as a continuous variable.

All families completed a demographic form. Children were categorized as meeting the EHDI 1–3–6 guidelines or not (coded as 1 or 0, respectively) on the basis of the parents’ report of whether the child received a hearing screen at birth, the age the child’s hearing loss was confirmed, and the age they started early intervention. Maternal level of education was also obtained from the demographic form and entered as a continuous variable (years of education completed).

Audiologic records were used to determine degree of hearing loss. Children were categorized into 2 groups based on their better-ear pure tone average: mild to moderate hearing loss (coded as 0) versus moderate-severe through profound hearing loss (coded as 1).

Quantity of parent talk directed to the child during the toddler and preschool years was based on 25-minute parent-child free-play language samples collected during a previous study when the current participants were 12 to 51 months of age (mean age = 32.2 months). All language samples were obtained in an environment that was familiar to the child and parent. Specifically, samples obtained during the 12- to 39-month period were collected in the child’s home, and those gathered when the child was 40 to 51 months of age were taped in a quiet room at the child’s school or local library. All language samples were gathered before collection of the pragmatics scores that were examined in the predictive model of this study. One to 6 language samples were collected from each child (mean = 2.25). See Table 4 for details. The samples were transcribed and analyzed by using the Systematic Analysis of Language Transcripts.^36^ The number of words produced by the parent was averaged across the samples for each participant. To account for the fact that a small number of samples were slightly shorter than 25 minutes, parent talk was quantified in terms of parent words per minute.

The proportion of children in each category of the dichotomous predictor variables are presented in Table 2. The means and SD of the continuous predictor variables and the outcome variable are summarized in Table 5.

#### Statistical Analyses

For the primary analyses, investigators used a 2-level hierarchical linear model with a random intercept to evaluate intraand inter-individual differences in pragmatics scores. Age was centered on 84 months, whereas all other predictors were centered on their corresponding mean. This procedure resulted in the overall intercept being the adjusted mean predicted pragmatics score at 84 months of age.

Each child’s observed growth was modeled at level 1, with the child’s age in months at the time of an assessment as the level 1 predictor. Nonvarying child and parent characteristics (eg, maternal education) were then used as level 2 predictors of both a child’s intercept (ie, do predicted pragmatics scores at 84 months of age vary on the basis of child and parent characteristics) and growth (ie, does growth in pragmatics scores over time vary on the basis of the child and parent characteristics). Preliminary screening and regression diagnostics revealed that the skew and kurtosis of the residuals were small and nonsignificant, with the absolute magnitude of all residuals <3. An examination of residual plots versus all predictors found no heteroscedasticity. The maximum variance inflation factor across all predictors was <1.11, suggesting minimal multicollinearity.

## RESULTS

### Preliminary Analyses

In Table 6, we present a correlation matrix of the child-level (ie, level 2) predictor variables. Correlations were generally nonsignificant or modest, with no correlations greater than *r* = 0.22.

### Primary Analyses

An initial “null” model simply examined variation in pragmatics scores across children and time with no predictors included. Adding the level 1 predictor of child age at the time of each assessment accounted for 52.6% of the level 1 variance in pragmatics scores (ie, the within-child variability in scores). The 5 level 2 variables were then added as predictors of the intercept term: in essence, assessing whether these child and parent characteristics had an overall impact on pragmatics performance. These 5 level 2 variables (nonverbal IQ, degree of hearing loss, mother’s level of education, parent words per minute, and meeting EHDI 1–3–6 guidelines) accounted for 42.4% of the level 2 mean child-to-child variance in pragmatics scores. Subsequent analyses were also used to examine whether the level-2 variables predicted growth in pragmatics skills (ie, whether they predicted the level 1 age coefficient). None were statistically significant, and so these were not included in the final model. The final model is presented in Table 7.

As reflected in Table 7, adjusting for all other variables, the predicted mean pragmatics score at 84 months of age was 8.31, reflecting that 83.1% of the items on the pragmatics scale were produced by the children using sentences of 4 words or more. Age was also highly related to performance, with a mean increase of 0.15 points per month over the course of the study (*b* = 0.15, *P* < .001). Focusing on the child-level characteristics, degree of hearing loss was related to pragmatics scores, with children diagnosed with moderate or severe to profound hearing loss performing the equivalent of 0.28 SD lower than those with mild or moderate hearing loss (*b* = −1.08, *P* = .034). Leiter full-scale IQ scores also were related to pragmatics scores, with an increase of 0.08 in a child’s score for each 1-point increase in Leiter IQ (*b* = 0.08, *P* < .001). In essence, a 15-point increase in Leiter IQ scores (ie, close to 1 SD in this sample) was associated with a 0.30 SD increase in pragmatics performance.

“Parent words per minute” was also a significant predictor of pragmatics scores (*b* = 0.04, *P* = .003). In this case, a 1 SD increase in the number of words spoken by the parent was associated with the equivalent of a 0.19 SD increase in predicted pragmatics performance. Finally, meeting the EHDI 1–3–6 guidelines was also a significant predictor of pragmatics scores (*b* = 1.01, *P* = .043). Specifically, children who met the EHDI 1–3–6 guidelines had pragmatics scores that were 0.26 SD higher than those who did not meet these guidelines.

## DISCUSSION

In the current study, we investigated longitudinal development and predictors of pragmatic language performance in 124 children with bilateral hearing loss from 4 to 7 years of age. Like children with typical hearing,^3,5^ as a group, there was significant growth in pragmatic language abilities as a function of age. Although this is encouraging, by the age of 7, the children’s mean predicted score was 83% of the items on the checklist, whereas, using the same instrument as in the current study, Goberis et al^5^ reported that by 4 years of age, children who were hearing mastered 95% of the items. Researchers in other studies have confirmed that children who are deaf or hard of hearing demonstrate particular difficulties with the pragmatic aspect of language,^4,6,7^ even when other areas of language are within the normal range.^6^ This discrepancy between the abilities of children with and without hearing loss provides motivation to examine factors that place children who are deaf or hard of hearing at increased risk of pragmatic language delay.

In the current study, predictors of better pragmatic language outcomes included (1) meeting EHDI 1–3–6 guidelines, (2) greater quantity of parent talk, (3) higher nonverbal IQ, (4) lesser degrees of hearing loss, and (5) higher maternal level of education. The relationships among these variables and between the predictor and outcome variables are likely complex, making the relative causal impacts difficult to ascertain without further research involving larger samples. In addition, although these 5 variables accounted for 42% of the variance in pragmatic skills, there are certainly additional uncontrolled factors (eg, level of parent involvement, quality of the children’s elementary educational setting, etc) that also contributed to pragmatic language outcomes.

The findings of the current study are consistent with those of previous studies of children who are deaf or hard of hearing in which researchers found an association between other aspects of language (eg, vocabulary, syntax, etc) and meeting EHDI 1–3–6 guidelines,^24^ nonverbal IQ,^28,37,38^ lesser degrees of hearing loss,^24–28^ and higher maternal education.^24,30,31^ With this study, we extend these findings to the pragmatic area of language.

In the current study, we indicate that timely identification of hearing loss and early intervention are critical for the optimal development of pragmatic skills. For context, the observed effect size for meeting EHDI 1–3–6 guidelines was on par with 4.32 years of additional maternal education or essentially equivalent to the difference between mild or moderate hearing loss and moderate-severe to profound hearing loss. In this study, all participants had been enrolled in Colorado Home Intervention Program, an early intervention program in which the providers have graduate-level training and expertise specific to both deafness and early childhood development. It is likely that the benefits of receiving intervention early are maximized when these services are provided by highly qualified professionals, and the positive impact of meeting EHDI guidelines may be less pronounced when early intervention is provided by less skilled personnel.

The amount of parent talk (quantified as the number of parent words directed to the child during free-play interactions) when the participants were younger (ages 1–4 years) also was a significant predictor of the children’s pragmatic language ability when they were 7 years of age. This finding is consistent with studies of children who are hearing in which greater quantities of parent talk have been associated with better child vocabulary and other language outcomes.^32–34,39^ Given previous findings that maternal education and other indicators of socioeconomic status are related to amount of parent input,^33,39^ it is likely that, within the current study, a complex relationship exists between amount of parent talk, maternal level of education, and child language outcomes.

The significant associations found in previous studies between the quantity of parent talk and child language skills has prompted researchers to examine if amount of parent talk serves as a proxy for the quality of parental input. Coady et al^40^ found that higher quantity of parent talk during parent-child interactions was associated with the parents’ use of more diverse vocabulary, longer utterances, and the use of a variety of child languageenhancing strategies including expansions and imitations of the child’s utterances. Hart and Risley^33^ also posit that quantity of speaking is linked to the amount of quality features in the language used. Authors of these studies, in combination with the current investigation, suggest that early intervention focused on increasing both the quantity and quality of parents’ communication with their child who is deaf or hard of hearing may result in improved child language outcomes.

## CONCLUSIONS

In this study, a variety of variables were found to predict pragmatic language abilities of children who are deaf or hard of hearing at 7 years of age. Of these, 2 factors (meeting the EHDI 1–3–6 guidelines and amount of parent talk) can be positively influenced by pediatricians and other health care professionals, such as audiologists, speech-language pathologists, and otolaryngologists. Specifically, pediatricians, otolaryngologists, and audiologists can support the goals of EHDI by urging families whose child has referred on a newborn hearing screen to seek diagnostic hearing evaluation and enrollment into early intervention as soon as possible. One of the primary benefits of early intervention is its role in providing parents with strategies that will enhance both the quantity and quality of their communication with their child, which is another factor found in this study to predict better pragmatic language skills. This strong association between amount of child-directed parent talk in the first 4 years of life and pragmatic language outcomes at 7 years of age emphasizes the need for a team approach with both early interventionists and pediatricians encouraging parents to talk to their children as much as possible.

## Figures and Tables

**TABLE 1:** Participant and Family Demographic Characteristics

Characteristic	Percentage of Participants
Gender	
Male	51
Female	49
Ethnicity	
Non-Hispanic	83
Hispanic	17
Race	
White	87
African American or Black	2
Asian	5
Mixed race	6
Communication mode used with the child	
Primarily spoken language	84
Spoken language only	41
Spoken with very occasional use of sign	43
Sign language plus spoken language	16
Mother’s highest educational degree	
Less than high school	3
High school	33
Vocational	7
Associate	9
Bachelor	32
Graduate	16

**TABLE 2:** Characteristics of the Participants’ Hearing Loss

Characteristic	Percentage of Participants
Degree of hearing loss	
Mild to moderate	36
Mild (26–40 dB HL)	20
Moderate (41–55 dB HL)	16
Moderate-severe to profound	63
Moderate-severe (56–70 dB HL)	20
Severe (71–90 dB HL)	20
Profound (>90 dB HL)	24
Met EHDI 1–3–6 guidelines	
Yes, met guidelines	46
No, did not meet guidelines	53
Type of amplification used	
Hearing aids	56
Cochlear implant	29
Bone conduction hearing aid	5
Hearing aid plus cochlear implant	10

Degree of hearing loss was determined on the basis of the better-ear pure tone average (ie, the average of hearing thresholds at 500, 1000, and 2000 Hz). HL, hearing loss.

**TABLE 3:** Number and Percentage of Participants Completing 1, 2, 3, or All 4 Assessments

No. Assessments Completed	No. Participants	Percentage of Participants
1	30	24.2
2	35	28.2
3	34	27.4
4	25	20.2

**TABLE 4:** Number and Percentage of Participants Completing Various Numbers of Language Samples

No. Language Samples Completed	No. Participants	Percentage of Participants
1	57	46
2	23	18.5
3	16	12.9
4	13	10.5
5	14	11.3
6	1	0.8

**TABLE 5:** Mean and SD of Continuous Variables

Variable	Mean	SD	*N*
Pragmatics score (scaled 0–10)	5.5	3.9	302
Chronological age, mo	65.7	13.3	302
Leiter full-scale IQ score	103.1	16.8	124
Parent words per min	53.5	16.8	124
Mothers’ education, y	14.5	2.6	124

**TABLE 6:** Correlation of Level 2 Child-Level Predictors (*n* = 124)

	1	2	3	4	5
Leiter nonverbal IQ	—	—	—	—	—
Mothers’ education, y	0.15	—	—	—	—
Parent words per min	0.09	0.20*	—	—	—
Degree of hearing loss^a^	−0.02	−0.12	−0.21*	—	—
Meets EHDI 1–3–6^b^	0.22*	0.18*	0.002	0.06	—

— , not applicable.

a Degree of hearing loss (0 = mild to moderate; 1 = moderate or severe to profound).

b Meets EHDI 1–3–6 guidelines (0 = does not meet EHDI; 1 = meets EHDI).

* *P* < .05, 2-tailed.

**TABLE 7:** Final 2-Level Model Predicting Pragmatics Scores

Effect	B	SE	*P*	95% CI
Intercept	8.31	0.30	<.001	(7.73 to 8.90)
Chronological age, mo^a^	0.15	0.01	<.001	(0.13 to 0.17)
Leiter full-scale IQ score	0.08	0.01	<.001	(0.05 to 0.11)
Degree of hearing loss^b^	−1.08	0.50	.034	(−2.07 to −0.08)
Parent words per min	0.04	0.01	.003	(0.02 to 0.07)
Mothers’ education, y	0.23	0.10	.017	(0.04 to 0.42)
Met EHDI 1–3–6 guidelines	1.00	0.49	.043	(0.03 to 1.97)

CI, confidence interval.

a Age was centered on 84 mo.

b Degree of hearing loss (0 = mild or moderate; 1 = moderate-severe to profound).

## WHAT’S KNOWN ON THIS SUBJECT:

Previous researchers have supported the benefits of meeting Early Hearing Detection and Intervention 1–3–6 guidelines on vocabulary development in children who are deaf or hard of hearing from 1 to 3 years of age.

## WHAT THIS STUDY ADDS:

With this study, we extend these findings to the pragmatic area of language and to children through 7 years of age. We also highlight the importance of child-directed parent talk during early childhood on later development of pragmatic language skills.

## ABBREVIATIONS

1–3–6: hearing screen by 1 month, identification of hearing loss by 3 months, and intervention by 6 months of age
EHDI: Early Hearing Detection and Intervention
IQ: intelligence quotient
